# Omega–3 Long-Chain Fatty Acids in the Heart, Kidney, Liver and Plasma Metabolite Profiles of Australian Prime Lambs Supplemented with Pelleted Canola and Flaxseed Oils

**DOI:** 10.3390/nu9080893

**Published:** 2017-08-17

**Authors:** Don V. Nguyen, Van H. Le, Quang V. Nguyen, Bunmi S. Malau-Aduli, Peter D. Nichols, Aduli E. O. Malau-Aduli

**Affiliations:** 1 Animal Genetics and Nutrition, Veterinary Sciences Discipline, College of Public Health, Medical and Veterinary Sciences, Division of Tropical Health and Medicine, James Cook University, Townsville, QLD 4811, Australia; donviet.nguyen@my.jcu.edu.au (D.V.N.); vanhung.le@my.jcu.edu.au (V.H.L.); quang.nguyen2@my.jcu.edu.au (Q.V.N.); peter.nichols@csiro.au (P.D.N.); 2 National Institute of Animal Science, Thuy Phuong, Bac Tu Liem, Hanoi 129909, Vietnam; 3 College of Economics and Techniques, Thai Nguyen University, Thai Nguyen 252166, Vietnam; 4 College of Medicine and Dentistry, Division of Tropical Health and Medicine, James Cook University, Townsville, QLD 4811, Australia; bunmi.malauaduli@jcu.edu.au; 5 CSIRO Oceans & Atmosphere, P.O. Box 1538, Hobart, TAS 7001, Australia

**Keywords:** prime lamb, oil supplementation, visceral organs, *n*-3 LC-PUFA, plasma metabolites

## Abstract

The objective of the study was to ascertain whether human health beneficial omega–3 long-chain (≥C_20_) polyunsaturated fatty acid (*n*-3 LC-PUFA) content in heart, kidney and liver can be enhanced by supplementing prime lambs with graded levels of canola and flaxseed oil. Health status of the lambs, as a consequence of the supplementation, was also investigated by examining their plasma metabolites. Sixty purebred and first-cross lambs were allocated to one of five treatments of lucerne hay basal diet supplemented with isocaloric and isonitrogenous wheat-based pellets without oil inclusion (Control) or graded levels of canola oil at 2.5% (2.5C), 5% (5C), flaxseed oil at 2.5% (2.5F) and 5% (5F) in a completely randomised design. Pre-slaughter blood, post-slaughter kidney, liver and heart samples were analysed for plasma metabolite and fatty acid profiles. Summations of docosapentaenoic acid and docosahexaenoic acid, and total *n*-3 LC-PUFA were enhanced in the liver and kidney of 5F supplemented lambs with a marked decrease in *n*-6/*n*-3 ratio and significant breed differences detected. There were generally no deleterious impacts on animal health status. A combination of 5% oil supplementation and lamb genetics is an effective and strategic management tool for enhancing *n*-3 LC-PUFA contents of heart, kidney and liver without compromising lamb health.

## 1. Introduction

The Australian Guide to Healthy Eating and Australian Dietary Guidelines [1] promote health and wellbeing by providing scientific evidence based dietary advice to reduce the risk of high cholesterol, high blood pressure, obesity, type 2 diabetes, cardiovascular disease and cancers. Therefore, consumers have become more aware and concerned about the relationship between dietary intake and health as their health consciousness increases. High levels of saturated fatty acids (SFA) and low content of polyunsaturated fatty acids (PUFA) in red meat have been implicated in the increased incidence of chronic diseases, especially cardiovascular diseases, diabetes and cancers [2,3]. Enser et al. [4] reported that lamb contains a higher fat percentage than beef and pork, and lower levels of PUFA in comparison with pork. Therefore, reducing fat content and modifying the fatty acid profile of lamb edible products have been warranted [5] and received much attention (see reviews by De Brito et al. [6] and Alvarenga et al. [7]).

The main way of improving the PUFA content in ruminants is the supplementation of PUFA enriched plant oils in their diets [8,9]. Canola and flaxseed oils, which contain an abundance of α-linoleic acid (ALA, 18:3*n*-3) [10,11], have been of recent interest in numerous nutritional trials in order to mainly improve *n*-3 LC-PUFA contents including eicosapentaenoic acid (EPA, 20:5*n*-3), docosapentaenoic acid (DPA, 22:5*n*-3) and docosahexaenoic acid (DHA, 22:6*n*-3) in sheep meat [12,13,14,15]. Genetic management of livestock for enhancing *n*-3 LC-PUFA content through selective breeding provides an alternative to nutritional manipulation and it is a cumulative and long-term approach. Several studies have been undertaken to investigate the impacts of different breeds on fatty acid profiles [16,17,18]. However, these investigations were only limited to the effect of breed on muscle fatty acid composition. In many countries, the consumption of non-carcass components, for instance heart, liver and kidney is very common. Furthermore, offal such as various organs can be a cheap source of proteins, minerals and vitamins [19,20] and play an important role in processed product formulations [20]. Liver and kidney are typically used in processed food products, such as pies and pasties which may not necessarily be healthy. Thus, lamb edible products include not only meat, but also these visceral organs. Meat and Livestock Australia (MLA) can strategically promote and encourage the direct intake and export of such offal organs instead of processed “junk food” if there were science-based empirical data suggesting that these offal organs contained health-claimable levels of *n*-3 LC–PUFA. The effects of different lipid sources and supplemented levels on altering the fatty acid profiles of liver and kidney in cattle and goat have been documented [5,21]. Kim et al. [22] also reported the variations in fatty acid composition in liver of Katadhin Dorper lambs fed 4.0% oil supplements with different *n*-6/*n*-3 fatty acid ratio. Nonetheless, studies investigating fatty acid profiles of heart, liver and kidney in prime lambs influenced by both dietary canola and flaxseed oil supplementation and breed are scanty and have not been undertaken under pasture-based production system.

Knowledge of haematological metabolite concentrations is valuable in understanding the individual health status and productivity of lambs [23]. Hence, quantifying the changes in plasma metabolite concentrations in lambs due to feed supplements and genetics is essential [24], especially the plasma metabolite profiles of prime lambs supplemented with graded levels of canola and flaxseed oil. The primary objective of this study was to investigate the effects of graded levels of canola and flaxseed oil supplementation to purebred Merino and first-cross prime lambs on heart, liver and kidney fatty acid profiles, including absolute contents, and the plasma metabolites. The secondary aim was to evaluate the interactions of supplementation level with breed.

## 2. Materials and Methods

### 2.1. Location and Animals

This research was conducted at the Cressy Research and Demonstration Station, Cressy, Tasmania, Australia, between June and August 2014. The experimental design and procedures were approved by the University of Tasmania Animal Ethics Committee (Permit No. A13839). The study was also in accordance with the 1993 Tasmania Animal Welfare Act and the 2013 Australian Code of Practice for the Care and Use of Animals for Scientific Purposes.

Sixty weaned ewe (*n* = 30) and wether (*n* = 30) prime lambs (seven months of age, mean 33.4 kg liveweight) were used in this feeding trial. The lambs comprised 20 purebred Merinos (M × M), 20 Corriedale × Merino (C × M) and 20 White Suffolk × Corriedale (W × C) first-cross lambs with equal number of ewe and wether lambs represented in each breed.

### 2.2. Experimental Design and Diets

A completely randomised experimental design balanced by breed and sex was utilised. Lambs were supplemented daily with 1 kg of isocaloric and isonitrogenous wheat-based pellets and allocated to one of five treatments of 12 lambs per group: no oil inclusion (Control); 2.5% canola oil (2.5C); 5% canola oil (5C); 2.5% flaxseed oil (2.5F) and 5% flaxseed oil (5F) on dry matter basis for 7 weeks after a three-week adaptation period. Lambs had *ad libitum* access to the basal diet of lucerne hay and clean water. Residual feed leftover was removed and weighed prior to fresh feed being offered to experimental lambs at 0900 h.

### 2.3. Blood and Visceral Organ Sampling

At the end of the feeding trial, blood samples were collected using jugular venipuncture. The lambs were organised for blood sample collection in the cool hours of the morning. Individual lambs within each treatment group were gently restrained in a relaxed sitting position with one researcher holding their heads ensuring they were comfortably upright and stable on flat ground to minimise individual animal variation and stress. These were stored in tubes containing heparin, immediately chilled in an esky containing ice and later centrifuged at 3000 rpm for 20 min at 4 °C. Plasma sub-samples were taken and stored at −20 °C for subsequent laboratory analysis.

After collecting blood samples, the lambs were walked to an adjacent commercial abattoir (100 m) and fasted overnight with water available in lairage. They were slaughtered the next day following Meat Standards Australia regulations. Heart, kidney and liver samples were taken immediately after evisceration. All samples were vacuum-sealed, code-labelled and stored at −20 °C until fatty acid analysis.

### 2.4. Feed Chemical Analysis

Concentrate pellet and lucerne hay samples were collected on days 0, 25 and 49 of the experimental period and kept at −20 °C for subsequent analyses. At the end of the experiment, the samples were defrosted; the three replicates for each sampling day were pooled and ground through a 1 mm screen. Samples were dried in triplicates in a fan-forced oven to a constant weight at 65 °C to determine dry matter (DM) content. Total Nitrogen (N) was quantified using an elemental analyser (PE2400 Series II; Perkin-Elmer Corp, Waltham, MA, USA), and multiplied by 6.25 to estimate crude protein (CP) content. Ether extract (EE) was determined using an ANKOM fat/oil extractor (ANKOM^XT15^; ANKOM Technology, Macedon, NY, USA). Acid detergent fibre (ADF) and neutral detergent fibre (NDF) contents were measured using an ANKOM fibre analyser (ANKOM^220^; ANKOM Technology, Macedon, NY, USA). Ash content was quantified by combusting the samples in a furnace at 550 °C for 5 h. Organic matter (OM) was computed as OM = 100 − Ash. Non-fibrous carbohydrates (NFC) was calculated as NFC = 100 − (CP + NDF + EE + Ash) [25]. Metabolisable energy (ME) was estimated using a near infrared reflectance spectroscopy method [26].

### 2.5. Fatty Acid and Plasma Metabolite Analyses

The total lipid extraction and fatty acid (FA) profile analysis of feed and visceral samples were undertaken at the Commonwealth Scientific and Industrial Research Organization (CSIRO), Oceans & Atmosphere, Hobart, Tasmania, Australia. The procedures were outlined in detail by Malau-Aduli et al. [27]. In brief, total lipids in 1 g of samples were solvent extracted using a modified Bligh and Dyer [28] protocol. CH_2_Cl_2_:MeOH:Milli-Q H_2_O (1:2:0.8 *v*/*v*) was used in a single-phase overnight process to extract lipids, followed by phase separation with CH_2_Cl_2_:saline Milli-Q H_2_O (1:1 *v*/*v*), and then rotary evaporated at 40 °C to obtain total lipids.

Aliquots of the total lipid extracts were methylated in methanol:dichloromethane (DCM):concentrated hydrochloric acid (10:1:1 *v*/*v*) for 2 h at 80 °C to produce fatty acid methyl esters (FAME). Glass test tubes fitted with Teflon-line screw caps (Brandon Scientific Glassblowing, Margate, TAS, Australia) were cooled and 1 mL of Milli-Q^®^ water added, along with 1.8 mL hexane: DCM (4:1 *v*/*v*). Tubes were vortexed and centrifuged at 2000 rpm for 5 min to break phase, with the upper, organic layer removed. This extraction step was repeated twice. The organic layer was reduced under a stream of nitrogen gas. DCM, with 0.1 mL of internal injection standard (19:0 FAME) was added. A 7890B gas chromatograph (GC) (Agilent Technologies, Palo Alto, CA, USA) equipped with an Equity™-1 fused silica capillary column (50 m × 0.32 mm internal diameter and 0.1-µm film thickness) (Supelco, Bellefonte, PA, USA), a flame ionisation detector, a split/splitless injector (Agilent Technologies, Palo Alto, CA, USA), and an Agilent Technologies 7683 B series autosampler was used to analyse the FAME samples. Fatty acid peaks were quantified by ChemStation software (Agilent Technologies, Palo Alto, CA, USA). GC-mass spectrometry (GC/MS) analyses were undertaken of selected samples to confirm FA identities and was performed using a Thermo Scientific 1310 GC coupled with a TSQ triple quadrupole (Thermo-Fisher Scientific, Milan, Italy). Samples were injected using a Tripleplus RSH auto sampler with a non-polar HP-5 Ultra 2 bonded–phase column (50 m × 0.32 mm i.d. × 0.17 µm film thickness; Agilent Technologies, Palo Alto, CA, USA). The HP-5 column was of similar polarity to the column used for GC analyses. The initial oven temperature of 45 °C was held for 1 min, followed by temperature programming at 30 °C per min to 140 °C then at 3 °C per min to 310 °C where it was held for 12 min. Helium was used as the carrier gas. Mass spectrometer operating conditions were: electron impact energy 70 eV; emission current 250 µA, transfer line 310 °C; source temperature 240 °C; scan rate 0.8 scan/sec and mass range 40–650 Da. Mass spectra were acquired and processed with Thermo Scientific XcaliburTM software (Waltham, MA, USA).

Fatty acid profiles comprised percentage (g/100 g of total FA (TFA) or %TFA) and content (mg/100 g of wet tissue). Fatty acid percentages were qualitatively calculated from FA area output: FA% = (area of individual FA) × (100)/(total FA area). Fatty acid content was computed as FA (mg/100 g) = (Total lipid percentage) × 0.916 × ((FA%)/100) × 1000 [29], with 0.916 as a lipid conversion factor [30] as cited by Clayton [29].

Plasma sub-samples were analysed for metabolite concentrations at the Animal Health Laboratory of the Tasmanian Department of Primary Industries, Parks, Water and Environment (DPIPWE), Launceston, Tasmania, Australia. Cholesterol, urea, calcium, magnesium, beta-hydroxybutyrate (BHB) and glucose were analysed on a Konelab 20XTi Clinical Chemistry Analyser (Thermo Scientific, Waltham, MA, USA).

### 2.6. Statistical Analysis

All data were analysed using Statistical Analysis System (SAS 2014, SAS Institute, Cary, NC, USA). Summary statistics including means and standard errors were computed and scrutinised for any erroneous data entry prior to running an analysis of variance (ANOVA). Since lambs were repeatedly weighed every week to compute average daily gains; feed samples taken on days 0, 25 and 49 of the experimental period and offal analysis was based on three replicates per organ, the need for a repeated measures analysis of variance model was warranted. The data were fitted into a repeated measures general linear model (PROC GLM) with oil supplementation, lamb breed, and their interaction as fixed effects, and total lipid percentages, FA profiles and plasma metabolites as dependent variables. Significant differences and mean separations at the *p* < 0.05 threshold were performed using Tukey′s probability pairwise comparison tests.

## 3. Results

### 3.1. Feed Ingredients, Chemical Compositions and Fatty Acid Profiles

The ingredients, chemical compositions and fatty acid profiles of the experimental pellets and lucerne hay are given in Table 1. The major carrier ingredient in the pellets was wheat (465–551 g/kg). The DM, CP, EE and ME contents, and other chemical compositions were relatively similar among the five treatment pellets. Lucerne hay had higher CP, NDF, ADF contents, and lower EE and ME contents than the pellets.

The prominent unsaturated fatty acids in pellets were 18:2*n*-6 and 18:1*n*-9, while 18:2*n*-6 and 18:3*n*-3 were the highest unsaturated fatty acids in lucerne hay. The control pellets had higher PUFA composition (48.4 g/100 g FA), but lower PUFA/SFA and *n*-6/*n*-3 ratios (2.0 and 11 respectively) compared to the oil supplemented pellets. Lucerne hay had low PUFA/SFA and *n*-6/*n*-3 ratios (0.9). EPA, DHA and DPA were not detected in the pellets and lucerne hay.

### 3.2. Heart Fatty Acid Profile

The inclusion of 5% flaxseed oil in pellets significantly reduced the *n*-6/*n*-3 ratio and 17:0 percentage (*p* < 0.05; Table 2) in comparison with the control pellets. However, oil supplementation did not result in any significant differences in the other FA compositions, PUFA contents and total lipid content of heart tissues.

Lamb breed significantly affected some FA compositions (*p* < 0.05; Table 2). Purebred Merinos had higher 17:0 (1.1 g/100 g FA) and total MUFA (26.7 g/100 g FA) compositions than W × C lambs (0.9 g/100 g FA and 23.8 g/100 g FA respectively). In contrast, the DPA and total *n*-6 PUFA percentages of W × C lambs were higher than those of M × M. There were no significant differences in PUFA contents and total lipid content of heart among the breeds.

**Figure 1:**
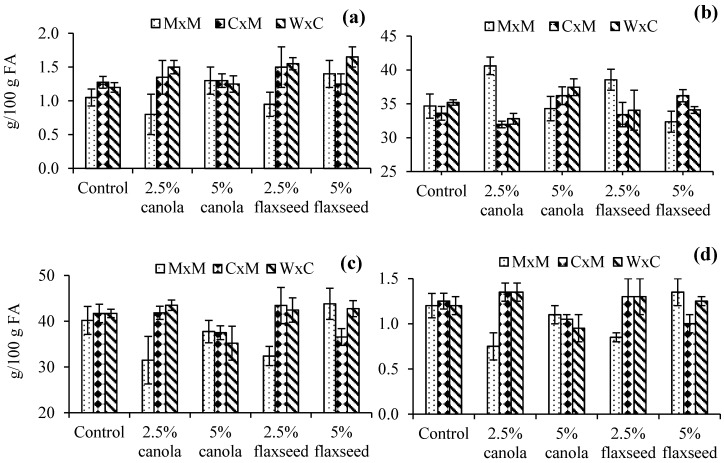
Interaction effects on: (**a**) docosapentaenoic acid, (**b**) total SFA, (**c**) total PUFA compositions and (**d**) PUFA/SFA ratio of lamb hearts (M × M: Merino × Merino; C × M: Corriedale × Merino; W × C: White Suffolk × Corriedale).

Significant interactions between oil addition and lamb breed on some heart FA compositions and PUFA/SFA ratio were detected (Figure 1). First-cross W × C lambs recorded the highest DPA composition (1.7 g/100 g FA) when they were fed pellets containing 5% flaxseed oil, while the lowest DPA composition was observed in M × M lambs fed 2.5% canola oil pellets (0.8 g/100 g FA) (Figure 1a). Furthermore, M × M lambs fed 2.5% canola oil pellets had higher total SFA composition than crossbred lambs offered the same pellets (Figure 1b), and contained lower total PUFA than crossbred lambs fed the same pellets and M × M and W × C lambs offered 5% flaxseed oil pellets (Figure 1c). Consequently, the PUFA/SFA ratio of M × M lambs fed pellets containing 2.5% canola oil was significantly lower than that of crossbred lambs fed the same pellets, and M × M lambs fed 5% flaxseed pellets (Figure 1d).

### 3.3. Liver Fatty Acid Profile

The lambs fed pellets containing 5% flaxseed oil had significantly higher liver DHA (3.4 g/100 g FA) and total *n*-3 PUFA (11.5 g/100 g FA) percentages (*p* < 0.05; Table 3) than the lambs offered the control pellets (2.4 g/100 g FA and 9.2 g/100 g FA respectively). The inclusion of 5% flaxseed oil in pellets also reduced 16:0 and 17:0 compositions, and *n*-6/*n*-3 ratio in lamb livers compared to the control pellets. It was apparent that the livers contained high levels of EPA + DHA (148.1–206.9 mg/100 g wet tissue) and total *n*-3 LC-PUFA (270.5–379.8 mg/100 g wet tissue). However, these contents were not influenced by oil supplementation. Lamb breed did not affect the total lipid percentage and FA profile of liver. There were no significant interactions between oil inclusion and lamb breed on liver total lipid percentage and FA profiles.

### 3.4. Kidney Fatty Acid Profile

Supplementing flaxseed oil in the pellets resulted in increases in the EPA and DPA compositions of lamb kidney compared to the control pellets (*p* < 0.05; Table 4). Furthermore, the kidneys of lambs fed pellets containing flaxseed oil were higher in total *n*-3 PUFA composition than those of lambs from the control and 2.5% canola oil groups. The *n*-6/*n*-3 ratio in kidneys significantly reduced for lambs offered flaxseed oil pellets. Oil supplementation significantly reduced the 17:0 composition of lamb kidney. The incorporation of 5% both canola and flaxseed oils in the pellets reduced 22:5*n*-6 content, while there were not significant differences in other PUFA contents including EPA + DHA and total *n*-3 LC-PUFA.

It was observed that lamb breed influenced variations in some kidney fatty acids (*p* < 0.05). First-cross W × C lambs recorded the highest 20:4*n*-6 (14.2 g/100 g FA) and DPA (3.2 g/100 g FA) compositions. However, they had lower 17:0 and DHA percentages in liver than M × M and C × M lambs. The livers contained more than 60 mg of EPA + DHA and 100 mg of total *n*-3 LC-PUFA in 100 g wet tissue (Table 4). The DHA and EPA + DHA contents of M × M liver were higher than those of C × M liver. There were no significant differences in kidney total lipid percentage among the breeds. Significant interactions between oil inclusion and lamb breed on the total lipid percentage and FA profiles of kidney were not observed. Liver and kidney of prime lambs had high contents of EPA + DHA and total *n*-3 LC-PUFA. These visceral organs can be classified as good sources of omega–3. A marked decrease in *n*-6/*n*-3 ratio of the investigated organs was observed when 5% flaxseed oil was added to the diet of finishing lambs. Supplementing 5% flaxseed oil also increased the DHA and total *n*-3 PUFA compositions of liver. Addition of flaxseed oil resulted in increases in the EPA, DPA and total *n*-3 PUFA compositions of kidney. First-cross W × C lambs had higher DPA compositions of heart and kidney than M × M. Lamb breed affected the DHA and EPA + DHA contents of kidney and plasma glucose concentration. Significant interactions between oil addition and lamb breed on heart FA compositions and plasma glucose concentration were observed.

### 3.5. Plasma Metabolites

**Figure 2:**
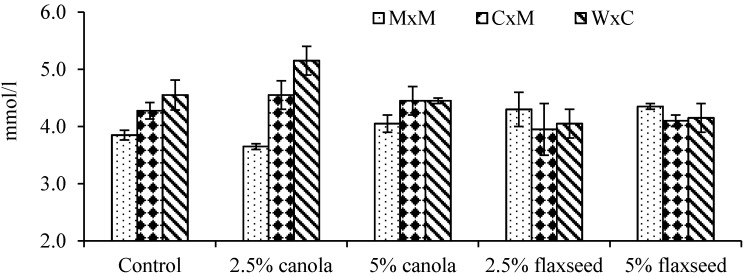
Variations in plasma glucose concentration as influenced by oil supplementation and breed interaction (M × M: Merino × Merino; C × M: Corriedale × Merino; W × C: White Suffolk × Corriedale).

Plasma metabolite concentrations of the experimental lambs are presented in Table 5. The glucose concentration of W × C lambs (4.5 mmol/L) was significantly higher (*p* < 0.05) than that of MxM lambs (4.0 mmol/L). Moreover, there was a significant interaction between oil supplementation and lamb breed on glucose concentration. First-cross W × C lambs fed pellets containing 2.5% canola oil had higher glucose concentration than M × M lambs fed the control and 2.5% canola oil pellets (Figure 2). Oil supplementation did not cause significant alteration in lamb plasma metabolites. However, the lamb urea concentration exceeded its normal ranges. No significant differences in plasma metabolite profiles between supplemented and control lambs imply that there were no obvious health disadvantages to the lambs as a result of oil inclusion.

## 4. Discussion

In Australia and New Zealand, food can be classified as a ‘source’ of *n*-3 PUFA if it contains at least 30 mg of EPA + DHA per standard serve and can be a ‘good source’ if it contains no less than 60 mg of EPA + DHA per standard serve [31]. A standard serve of red meat for Australian adults is reported to be 135 g [32,33]. Our study has demonstrated that hearts from lambs fed oil supplemented pellets could be a source of *n*-3 PUFA, although oil supplementation did not significantly influence EPA + DHA content. Furthermore, lamb kidneys and livers could be labelled as good sources of *n*-3 PUFA. Our research is in agreement with other studies that have assessed FA profile of edible by-products from lamb [27] and goat [20] and have reported that the total *n*-3 LC-PUFA content of liver and kidney were higher than that of heart.

The main traditional source of *n*-3 CL-PUFA is seafood including fish, crustaceans and molluscs, however, different types of seafood contain widely different amounts of *n*-3 LC-PUFA [34]. In a typical seafood serve size (150 g raw), white-fleshed Australian wild-caught fish contain approximately 350 mg *n*-3 CL-PUFA (233 mg/100 g raw), while shellfish and prawns have about 225 (150 mg/100 g raw) and 180 mg (120 mg/100 g raw) respectively [34]. Omega-3 LC-PUFA content of liver in the present study was generally higher than that of white-fleshed Australian wild fish. Furthermore, the contents of *n*-3 LC-PUFA in lamb kidney and prawns were similar. Thus, liver and kidney may be considered as alternative and ‘good sources’ of *n*-3 LC-PUFA.

Several epidemiological studies have demonstrated that consumption of the less studied *n*-3 LC-PUFA - DPA - is positively correlated with lower incidence of coronary heart diseases and platelet aggregation [35,36], improvement in lipid metabolism, and inhibition of inflammation [37]. Furthermore, Byelashov et al. [35] stated that DPA improves cognitive function and mental health. Red meat contains relatively high levels of DPA compared to fish [38] and plays a major part in many diets [39,40]. In the present study, it is evident that the content of DPA in heart, kidney and liver was higher than those of EPA and DHA. Howe et al. [41] stated that DPA contributes approximately 30% of total *n-*3 LC-PUFA in Australian diets. Therefore, it is being increasingly discussed that DPA should be included in total *n*-3 LC-PUFA intake [35,41,42]. In fact, the Australian and New Zealand governments have offered guidelines for DPA intake along with EPA and DHA [43]. Including DPA in *n*-3 LC-PUFA intake would boost the total *n*-3 LC-PUFA content of lamb to higher values [29,38] which is in agreement with our findings.

It is now widely accepted that lipid supplementation of ruminant diets is an effective approach to modify edible fats. Feeding canola oil or flaxseed oil is a straightforward way to increase *n*-3 LC-PUFA in ruminant meat [44]. Nguyen et al. [45] also concluded that supplementing 5% of these oils (via supplementation) in prime lamb diets increased *n*-3 LC-PUFA contents of *Longissimus* muscle. Indeed, the inclusion of 5% flaxseed oil in this study increased the relative composition and content of liver DHA and kidney DPA, and total *n*-3 PUFA compositions in both liver and kidney in comparison with the control diet. These increases could be due to the increase in the intake and the ruminal concentration of ALA increased in diet [5,45]. Dietary ALA is an important *n*-3 PUFA that is used as a precursor for the production of tissue *n*-3 LC-PUFA including EPA, DPA and DHA. Research has shown that ruminant *n*-3 LC-PUFA composition can be improved by increased dietary ALA intake from ALA-abundant vegetable oils [9,46]. Our findings are in agreement with Adeyemi et al. [5] who found significant increases in the EPA and total *n*-3 PUFA compositions of liver when supplementing up to 8% with a blend of 80% canola oil and 20% palm oil in goat diets. Similarly, Kashani et al. [47] investigated the FA compositions of different tissues in dual-purpose Australian lambs and concluded that liver EPA and total *n*-3 PUFA compositions were significantly influenced by *Spirulina* supplementation. Kim et al. [22] and Demirel et al. [48] also reported the *n*-3 LC-PUFA compositions of lamb liver increased when dietary ALA intake was increased. Therefore, our results in combination with previous studies indicate that tissue *n*-3 LC-PUFA compositions are influenced by the level of dietary ALA intake.

Ruminant FA profiles are also directly associated with the extensive biohydrogenation of dietary MUFA and PUFA conducted by ruminal microbes. In the rumen, ALA is hydrogenated to rumelenic acid (*cis*-9, *trans-*11, *cis-*15 18:3) and subsequent reduction via rumenic acid (*cis*-9, *trans-*11 18:2), then to vaccenic acid (*trans*-11 18:1) and finally to stearic acid (18:0) [49] with an extent of 85–100% [50]. Furthermore, Doreau and Ferlay [50] stated that lipid digestibility in the small intestine is independent of the level of FA intake. This could explain the absence of significant oil supplementation effects on the *n*-3 LC-PUFA contents of heart, liver and kidney in our study.

Our findings indicated that variations in the FA profiles of editable organs were influenced by lamb breed, a finding that can be summarised as the individual *n*-3 LC-PUFA compositions of heart and kidney differed between M × M and W × C lambs. These variations could be explained by differences occurring in fatty acid metabolism, lipogenesis and deposition arising from genetic variation, as indicated by previous studies [18,51]. This interpretation is supported by Sanudo et al. [52] who found Merino lambs had lower EPA and DPA compositions than coarse wool breeds. In contrast, Hoffman et al. [53] did not observe differences in the EPA and DPA compositions of cooked heart, liver and kidney between Merino and Dorper lambs. The possible explanation for the observed differences between our study and that of Hoffman et al. [53] is the status of investigated tissues. Tissue FA profiles could be changed during cooking [54,55]. In the present study, lamb breed did not affect total *n*-3 PUFA compositions and *n*-3 LC-PUFA contents, consistent with the report of Malau-Aduli et al. [27]. Various studies stated that the effects of variations in *n*-3 LC-PUFA due to lamb breed were much more complicated and of a lower magnitude than variations due to the nutritional background [27,32,56].

The results of our study found no significant effect of supplementing enriched omega–3 oils on plasma cholesterol concentration in contrast to Obeidat et al. [57] and Bhatt et al. [58] who concluded that increases in cholesterol occurred. The variations in forms and higher dosages of fat sources used in these previous studies could explain the observed differences. Various studies reported that lipid supplementation did not influence the glucose and urea concentrations of ruminant plasma [59,60,61]. In agreement with these studies, our results showed that the inclusion of canola oil or flaxseed oil did not significantly alter the plasma concentrations of glucose and urea. An explanation for the absence of significant differences in plasma metabolite concentrations could be related to the similarity of metabolisable energy components among the diets used in our study. Second, the oil levels used in this study were at or below 50 g/kg dry matter intake (DMI) to avoid the possibility of negative effects on DMI and nutrient digestibility [62].

In agreement with our results, Malau-Aduli and Holman [63] and Malau-Aduli et al. [64] found that differences in plasma glucose among lamb breeds were observed. In beef cattle, Stahlhut et al. [65] also reported Angus cows had higher plasma glucose concentrations than Simmental cows and concluded that plasma glucose concentration was influenced by average daily gain, milk production and FA profile of carcass. Differences in average daily gain [66] and FA profile [27,55] have been reported between purebred Merino and crossbred lambs, indicating a potential for differences in plasma glucose concentration between the breeds. The high concentration of plasma urea in the present study could be due to high crude protein levels in the diets. Butler [67] and Sunny et al. [68] stated that the high dietary intake of protein and the balance of protein fractions present in the rumen can result in increased blood concentrations of urea.

### Potential Human Health Implications for Consumption of Lamb Liver and Kidney

Nguyen et al. [45] reported the nutritional value, lipid percentage, fatty acid profile and sensory characteristics of visceral adipose and muscle tissues in these same 60 lambs used in the present study. They demonstrated that a standard serve (135 g) of meat produced from lambs supplemented with 5% oil contained more than 30 mg of EPA + DHA, reached the claimable ‘source’ level of *n*-3 LC-PUFA and significantly affected meat tenderness, juiciness and overall liking by human meat consumers. The finding in the current study that lamb liver and kidney also reached the claimable ‘source’ level of *n*-3 LC-PUFA has potential human health implications from the viewpoint of their EPA, DHA and DPA contents. In the *n*-3 synthesis pathway, DPA is an intermediary between EPA and DHA [69]. Many epidemiological studies in humans have demonstrated that DPA consumption is positively correlated with lower incidence of coronary heart diseases and lower platelet aggregation [36,70], and in hamsters, inhibition of inflammation and improvement in lipid metabolism [37]. Furthermore, Lim et al. [71] stated that DPA improves mental health and spinal cord injury in mice, but the roles of DPA in human health have been largely ignored, perhaps because it is a negligible component of commercial fish and oil products compared to EPA and DHA *n*-3 LC-PUFA [35,43]. DPA contributes approximately 30% of total *n*-3 LC-PUFA in human diets [41]. It can serve as a reservoir for EPA and DHA because it is either retro-converted to EPA or elongated to DHA [72,73]. Thus, some reports have suggested that DPA should be included in LC-PUFA intake [41,42]. Indeed, Australia and New Zealand have offered guidelines for DPA intake along with EPA and DHA [43]. Including DPA in *n*-3 LC-PUFA intake would boost the total *n*-3 LC-PUFA content produced in lamb meat to higher values [29].

## 5. Conclusions

The results clearly demonstrated that both canola and flaxseed oils can be effectively used in feedlot regimes in the prime lamb industry without any detrimental health effects. For prime lamb producers wanting to achieve better product quality, in terms of FA composition, during the 10-week intensive finishing phase, supplementing 5% flaxseed oil into the animal diets considerably improves the content of the health benefitting *n*-3 LC-PUFA in the edible visceral organs. On the basis of the favourable FA content findings presented, lamb liver and kidney could be consumed as alternative ‘good sources’ of *n*-3 LC-PUFA.

## Figures and Tables

**Table 1:** Ingredients, chemical compositions and fatty acid percentage of feeds.

Item	Pellet Feed ^1^					Lucerne Hay
	Control	2.5C	5C	2.5F	5F	
Ingredients (g/kg)						
Wheat	513	537	545	551	465	‒
Paddy rice	260	230	210	220	280	‒
Lupins	170	151	138	147	148	‒
Canola oil (mL/kg)	-	25	50	-	-	‒
Flaxseed oil (mL/kg)	-	-	-	25	50	‒
Salt	10	10	10	10	10	‒
Limestone	21	21	21	21	21	‒
Sheep premix	1	1	1	1	1	‒
Ammonium sulfate	12.6	12.6	12.6	12.6	12.6	‒
Acid buff	6.2	6.2	6.2	6.2	6.2	‒
Sodium bicarbonate	6.2	6.2	6.2	6.2	6.2	‒
Chemical compositions (% dry matter) ^2^						
Dry matter, (%)	89.8	90.2	87.9	90.5	89.4	89.6
Crude protein	14.7	14.5	14.4	14.5	14.5	17.4
NDF	23.8	23.5	23.9	23.7	23.3	46.5
ADF	9.2	9.3	8.9	9.5	9.0	30.9
NFC	50.5	49.9	47.8	50.5	50.7	27.4
Ether extract	3.0	4.6	5.7	4.2	5.1	2.4
Ash	8.0	7.5	8.2	7.1	6.4	7.2
ME (MJ/kgDM)	10.7	10.9	11.1	10.8	11.1	9.8
Fatty acid percentage (g/100 g total FA) ^3^						
14:0	0.2	0.5	0.6	0.2	0.2	0.6
15:0	0.1	0.1	0.1	0.1	0.1	0.4
16:0	18.2	16.9	16.5	19.1	19.8	29.6
17:0	0.1	0.1	0.2	0.1	0.1	0.7
18:2*n*-6	43.4	28.4	26.7	25.6	24.7	19.1
18:3*n*-3	3.5	3.6	4.3	4.9	7.2	18.8
18:1*n*-9	23.9	38.9	37.5	32.3	34.1	5.6
18:0	3.4	4.1	4.1	4.4	5.1	4.7
20:3*n*-6	0.3	0.4	0.4	0.4	0.5	0.4
20:4*n*-3	0.4	0.5	0.2	0.5	0.6	0.5
20:2*n*-6	0.1	0.1	0.2	0.1	0.1	0.1
20:0	0.5	0.8	0.7	0.7	0.8	1.5
∑SFA	24.1	23.0	25.0	26.7	28.7	47.3
∑MUFA	27.5	42.6	43.3	36.3	37.5	12.7
∑PUFA	48.4	34.4	31.7	37.0	33.8	40.0
PUFA/SFA	2.0	1.5	1.3	1.4	1.2	0.9
∑*n*-3 PUFA	3.9	4.1	4.8	5.5	7.9	20.5
∑*n*-6 PUFA	43.8	28.8	27.4	26	25.3	19.4
*n*-6/*n*-3	11.1	7.0	5.7	4.7	3.2	0.9

^1^ 2.5C: 2.5% canola oil; 5C: 5% canola oil; 2.5F: 2.5% flaxseed oil; 5F: 5% flaxseed oil; ADF: acid detergent fibre; MJ: mega joules; DM: dry matter. ^2^ NFC: non-fibrous carbohydrates (NFC = 100 − (crude protein (CP) + neutral detergent fibre (NDF) +ether extract (EE) + ash)); ME: metabolisable energy. ^3^ ∑SFA: total saturated fatty acid includes: 14:0, 15:0, 16:0, 17:0, 18:0, 20:0, 21:0, 22:0, 23:0, 24:0; ∑MUFA: total monounsaturated fatty acid includes: 14:1, 16:1*n*-9, 16:1*n*-7, 16:1*n*-5, 16:1*n*-13, 17:1*n*-8 + a17:0, 17:1, 18:1*n*-9, 18:1*n*-7, 18:1, 19:1, 20:1*n*-11, 20:1*n*-9, 20:1*n*-7, 20:1*n*-5, 22:1*n*-9, 22:1*n*-11, 22:1*n*-9, 24:1*n*-9; ∑PUFA: total polyunsaturated fatty acid includes: 18:3*n*-6, 18:2*n*-6, 18:3*n*-3, 20:4*n*-3, 20:4*n*-6, 20:5*n*-3, 20:3*n*-6, 20:2*n*-6, 22:5*n*-6, 22:6*n*-3, 22:5*n*-3, 22:4*n*-6, 24:6*n*-3, 24:5*n*-3; ∑*n*-3 PUFA: total omega–3 PUFA includes 18:3*n*-3, 20:5*n*-3, 20:4*n*-3, 22:6*n*-3, 22:5*n*-3; ∑*n*-6 PUFA: total omega–6 PUFA includes 18:3*n*-6, 18:2*n*-6, 20:4*n*-6, 20:3*n*-6, 20:2*n*-6, 22:5*n*-6, 22:4*n*-6.

**Table 2:** Effects of omega–3 oil supplementation and breed on heart fatty acid profile of prime lambs.

Item ^1^		Treatment				Breed ^2^			SEM ^3^	*p* Value ^4^		
	Control	2.5C	5C	2.5F	5F	M × M	C × M	W × C		T	B	T × B
Total lipid (g fat/100 g wet tissue)	2.6	2.7	2.8	2.9	2.4	2.8	2.7	2.5	0.09	0.35	0.23	0.16
Percentage (g/100 g total FA)												
14:0	0.6	0.6	0.8	0.6	0.5	0.6	0.7	0.5	0.05	0.68	0.33	0.24
15:0	0.2	0.2	0.2	0.2	0.2	0.2	0.2	0.2	0.01	0.36	0.94	0.30
16:0	12.9	12.5	13.1	12.9	12.7	12.9	12.9	12.7	0.22	0.93	0.63	0.06
17:0	1.1 ^a^	1.0 ^a,b^	1.0 ^a,b^	0.9 ^a,b^	0.8 ^b^	1.1 ^a^	1.0 ^a,b^	0.9 ^b^	0.03	0.04	0.02	0.07
18:2*n*-6	23.8	21.0	21.0	21.9	22.4	20.9	23.1	22.9	0.48	0.11	0.06	0.07
18:3*n*-3	2.0	1.8	2.5	2.1	2.7	2.5	2.1	1.7	0.18	0.53	0.24	0.56
18:1*n*-9	13.4	15.5	15.6	14.2	12.8	14.9	14.7	12.9	0.45	0.17	0.08	0.39
18:0	17.2	18.4	18.4	17.9	17.5	18.3	17.3	17.6	0.27	0.35	0.12	0.06
20:4*n*-6 (ARA)	5.6	6.2	4.3	5.8	5.2	5.1	4.8	6.5	0.33	0.33	0.06	0.06
20:5*n*-3 (EPA)	1.2	1.3	1.2	1.3	1.4	1.2	1.3	1.3	0.04	0.35	0.33	0.07
20:3*n*-6	0.5	0.4	0.4	0.4	0.5	0.4	0.4	0.5	0.01	0.22	0.37	0.44
20:4*n*-3	1.4	1.4	1.5	1.6	1.7	1.4	1.5	1.6	0.06	0.65	0.47	0.49
20:2*n*-6	0.1	0.1	0.1	0.1	0.1	0.1	0.1	0.1	0.01	0.81	0.77	0.91
20:0	0.2	0.2	0.2	0.2	0.2	0.2	0.2	0.2	0.01	0.99	0.74	0.87
22:5*n*-6	0.1	0.1	0.1	0.1	0.1	0.1	0.1	0.1	0.01	0.25	0.23	0.23
22:6*n*-3 (DHA)	0.6	0.7	0.7	0.7	0.7	0.7	0.6	0.7	0.03	0.85	0.56	0.72
22:4*n*-6	0.2	0.2	0.1	0.2	0.2	0.1	0.1	0.2	0.01	0.72	0.20	0.57
22:5*n*-3 (DPA)	1.1	1.1	1.2	1.3	1.4	1.2 ^b^	1.3 ^a,b^	1.4 ^a^	0.05	0.19	0.04	0.02
22:0	0.3	0.3	0.2	0.3	0.3	0.3	0.3	0.3	0.01	0.74	0.22	0.83
23:0	0.3	0.3	0.3	0.3	0.3	0.3	0.3	0.3	0.01	0.37	0.56	0.74
24:0	0.3	0.2	0.2	0.2	0.2	0.2	0.2	0.2	0.01	0.85	0.46	0.67
∑SFA	34.5	35.1	35.9	35.2	34.2	35.5	34.5	34.8	0.46	0.63	0.31	0.01
∑MUFA	24.3	26.3	27.3	25.3	24.8	26.7 ^a^	25.5 ^a,b^	23.8 ^b^	0.50	0.27	0.04	0.30
∑PUFA	41.2	38.6	36.8	39.5	41.0	37.8	40.0	41.4	0.87	0.30	0.08	0.03
PUFA/SFA	1.2	1.2	1.0	1.2	1.2	1.1	1.2	1.2	0.04	0.52	0.06	0.01
∑*n*-3 PUFA	6.5	6.4	6.9	6.6	7.9	7.0	6.7	6.7	0.25	0.36	0.90	0.26
∑*n*-6 PUFA	30.3	28.1	26.1	28.6	28.5	26.9^b^	28.7 ^a,b^	30.3 ^a^	0.68	0.17	0.02	0.06
*n*-6/*n*-3	4.8 ^a^	4.4 ^a,b^	4.1 ^a,b^	4.3 ^a,b^	3.6 ^b^	4.0	4.4	4.6	0.15	0.02	0.28	0.83
Content (mg/100 g wet tissue)												
18:3*n*-3	24.1	26.7	24.6	27.5	31.0	31.7	18.7	26.7	2.19	0.83	0.09	0.81
20:4*n*-6	77.1	101.7	59.9	68.8	71.0	68.6	60.4	99.0	7.44	0.55	0.08	0.58
EPA	13.7	15.7	15.4	18	20.5	16.1	13.1	20.3	1.37	0.64	0.07	0.56
22:5*n*-6	1.1	1.1	1.2	1.1	0.9	1.0	1.0	1.3	0.09	0.89	0.23	0.94
DHA	7.8	8.0	8.1	9.3	10.4	9.2	6.4	10.1	0.71	0.79	0.14	0.90
DPA	14.0	15.3	16.1	18.0	20.0	15.3	13.4	20.6	1.37	0.72	0.07	0.58
EPA + DHA	21.5	23.7	23.5	27.3	30.9	25.3	19.5	30.4	2.02	0.67	0.09	0.72
EPA + DHA + DPA	35.5	39.0	39.6	45.3	50.9	40.6	32.9	51.0	3.36	0.69	0.08	0.66

^1^ All definitions and abbreviations are as indicated in Table 1; FA: fatty acid; ARA: arachidonic acid; EPA: eicosapentaenoic acid; DPA: docosapentaenoic acid and DHA: docosahexaenoic acid; ^2^ M × M: purebred Merino; C × M: Corriedale and Merino crossbred; W × C: White Suffolk and Corriedale crossbred; **^3^** SEM: standard error of the mean; ^4^ T: treatment; B: breed; T × B: treatment × breed. Row means bearing different superscripts within a fixed factor differ significantly (*p* < 0.05). In all lamb tissues (Table 2, Table 3 and Table 4), low levels of trans 18:1 isomers and conjugated linoleic acid (CLA) isomers were also present.

**Table 3:** Liver fatty acid profile of prime lambs as influenced by omega–3 oil supplementation and breed.

Item ^1^		Treatment				Breed			SEM	*p* Value		
	Control	2.5C	5C	2.5F	5F	M × M	C × M	W × C		T	B	T × B
Total lipid (g fat/100 g wet tissue)	6.3	6.6	6.2	6.3	6.4	6.4	6.6	6.0	0.16	0.99	0.37	0.74
Percentage (g/100 g total FA) ^2^												
14:0	0.5	0.4	0.4	0.4	0.4	0.4	0.5	0.4	0.02	0.44	0.22	0.22
15:0	0.3	0.3	0.3	0.3	0.3	0.3	0.3	0.3	0.01	0.22	0.12	0.59
16:0	18.0 ^a^	17.4 ^a,b^	17.4 ^a,b^	17.3 ^a,b^	15.9 ^b^	17.2	17.3	17.2	0.28	0.03	0.85	0.47
17:0	1.4 ^a^	1.3 ^a,b^	1.3 ^ab^	1.3 ^a,b^	1.2 ^b^	1.4	1.3	1.3	0.03	0.02	0.24	0.56
18:2*n*-6	10.5	10.0	10.1	9.7	10.0	10.5	9.7	10.1	0.20	0.68	0.35	0.59
18:3*n*-3	2.0	2.3	2.5	2.5	2.7	2.4	2.5	2.1	0.10	0.17	0.53	0.66
18:1*n*-9	20.7	20.5	20.7	18.1	18.8	19.9	20.2	19.6	0.44	0.30	0.88	0.61
18:0	18.9	19.6	19.3	21.7	20.4	19.4	19.9	20.2	0.41	0.17	0.70	0.36
20:4*n*-6 (ARA)	4.2	4.6	3.1	4.4	4.5	3.7	4.7	4.0	0.33	0.73	0.57	0.77
20:5*n*-3 (EPA)	1.3	1.4	1.3	1.3	1.6	1.3	1.5	1.3	0.06	0.68	0.51	0.27
20:3*n*-6	0.7	0.7	0.7	0.6	0.6	0.7	0.7	0.7	0.03	0.50	0.86	0.07
20:4*n*-3	0.2	0.1	0.2	0.2	0.1	0.2	0.2	0.2	0.01	0.15	0.47	0.82
20:2*n*-6	0.1	0.1	0.1	0.1	0.1	0.1	0.1	0.1	0.01	0.27	0.30	0.54
20:0	0.1	0.1	0.1	0.1	0.1	0.1	0.1	0.1				
22:5*n*-6	0.2	0.2	0.2	0.2	0.2	0.2	0.2	0.2	0.01	0.33	0.26	0.70
22:6*n*-3 (DHA)	2.4 ^b^	2.5 ^b^	2.6 ^a,b^	2.9 ^a,b^	3.4 ^a^	2.9	2.6	2.5	0.13	0.03	0.50	0.18
22:4*n*-6	0.5	0.4	0.4	0.4	0.3	0.4	0.4	0.5	0.02	0.26	0.32	0.66
22:5*n*-3 (DPA)	3.3	3.1	3.4	3.4	3.7	3.1	3.3	3.6	0.12	0.64	0.24	0.47
22:0	0.2	0.2	0.2	0.2	0.2	0.2	0.2	0.2	0.01	0.62	0.99	0.92
23:0	0.4	0.4	0.4	0.3	0.3	0.4	0.4	0.4	0.01	0.71	0.83	0.21
24:0	0.3	0.3	0.3	0.3	0.3	0.3	0.3	0.3	0.01	0.80	0.84	0.06
∑SFA	40.8	41.3	41.4	44.1	40.6	41.2	41.6	41.8	0.54	0.23	0.90	0.12
∑MUFA	32.6	32.0	33.1	29.2	31.1	32.1	31.5	31.6	0.54	0.29	0.91	0.68
∑PUFA	26.6	26.7	25.5	26.7	28.3	26.7	26.9	26.6	0.59	0.92	0.98	0.47
PUFA/SFA	0.7	0.6	0.6	0.6	0.7	0.6	0.6	0.6	0.07	0.69	0.74	0.21
∑*n*-3 PUFA	9.2 ^b^	9.6 ^a,b^	9.7 ^a,b^	10.3 ^a,b^	11.5 ^a^	9.9	10.0	9.7	0.32	0.04	0.99	0.26
∑*n*-6 PUFA	16.5	16.3	14.8	15.6	15.9	15.9	16.0	15.9	0.40	0.73	0.96	0.67
*n*-6/*n*-3	1.8 ^a^	1.7 ^a,b^	1.6 ^a,b^	1.6 ^a,b^	1.4 ^b^	1.6	1.7	1.7	0.05	0.03	0.96	0.57
Content (mg/100 g wet tissue)												
18:3*n*-3	79.3	86.7	115.7	111.7	118.9	107.9	99.6	88.2	7.20	0.36	0.77	0.57
20:4*n*-6	186.0	176.6	135.6	225.2	202.5	177.0	192.6	184.2	19.89	0.85	0.96	0.88
EPA	50.9	53.5	58.2	64.8	71.6	56.9	61.2	56.0	4.73	0.86	0.90	0.59
22:5*n*-6	9.7	9.8	9.7	7.4	8.0	10.6	7.8	8.9	0.72	0.77	0.30	0.91
DHA	97.2	99.1	108.8	142.0	146.8	128.7	110.0	106.0	9.23	0.49	0.71	0.75
DPA	131.1	118.0	156.0	167.4	161.4	140.5	138.6	153.4	11.95	0.76	0.77	0.69
EPA + DHA	148.1	152.5	167.0	206.9	218.3	185.6	171.2	162.0	13.53	0.61	0.88	0.72
EPA + DHA + DPA	279.1	270.5	323.0	374.3	379.8	326.1	309.9	315.5	24.89	0.70	0.97	0.74

^1^ All definitions and abbreviations are as indicated in Table 1 and Table 2. FA: ^2^ fatty acid; Row means bearing different superscripts within a fixed factor significantly differ (*p* < 0.05).

**Table 4:** Variation in kidney fatty acid profile of prime lambs as influenced by omega–3 oil supplementation and breed.

Item ^1^		Treatment				Breed			SEM	*p* Value		
	Control	2.5C	5C	2.5F	5F	M × M	C × M	W × C		T	B	T × B
Total lipid (g fat/100 g wet tissue)	3.0	3.1	2.8	3.0	2.9	3.0	3.0	3.0	0.05	0.53	0.65	0.42
Percentage (g/100 g total FA)^2^												
14:0	0.4	0.3	0.3	0.3	0.3	0.3	0.3	0.3	0.02	0.44	0.77	0.65
15:0	0.3	0.3	0.3	0.3	0.3	0.3	0.3	0.3	0.01	0.68	0.58	0.68
16:0	17.0	17.2	16.5	16.4	16.0	16.6	17.0	16.3	0.19	0.53	0.47	0.94
17:0	1.2 ^a^	1.1 ^b^	1.1 ^b^	1.0 ^b,c^	0.9 ^c^	1.1 ^a^	1.1^a^	1.0^b^	0.02	0.00	0.02	0.07
18:2*n*-6	12.7	12.6	12.5	12.7	12.6	12.6	12.4	12.8	0.17	0.93	0.84	0.90
18:3*n*-3	1.1	1.1	1.2	1.2	1.5	1.2	1.3	1.1	0.06	0.22	0.49	0.11
18:1*n*-9	11.9	12.7	11.7	11.3	11.6	12.1	12.4	11.1	0.25	0.52	0.12	0.51
18:0	17.4	17.5	17.9	17.5	17.8	17.7	17.0	18.1	0.18	0.89	0.16	0.96
20:4*n*-6	13.8	13.7	13.2	12.7	12.1	12.5 ^b^	13.1 ^a,b^	14.2 ^a^	0.29	0.27	0.04	0.69
20:5*n*-3 (EPA)	1.9 ^b^	2.0 ^b^	2.3 ^a,b^	2.5 ^a^	2.8 ^a^	2.3	2.0	2.3	0.09	0.01	0.08	0.80
20:3*n*-6	0.9	0.8	0.8	0.9	0.8	0.8	0.8	0.9	0.03	0.86	0.11	0.72
20:4*n*-3	0.6	0.7	0.7	0.7	0.7	0.7	0.6	0.6	0.02	0.63	0.17	0.71
20:2*n*-6	0.3	0.3	0.3	0.3	0.3	0.3	0.3	0.4	0.01	0.91	0.65	0.27
20:0	0.3	0.3	0.3	0.3	0.3	0.3	0.3	0.3	0.01	0.30	0.29	0.32
22:5*n*-6	0.1	0.1	0.1	0.1	0.1	0.1	0.1	0.1	0.01	0.65	0.79	0.85
22:6*n*-3 (DHA)	2.3	2.3	2.4	2.4	2.4	2.5 ^a^	2.4 ^a^	2.1 ^b^	0.07	0.88	0.03	0.11
22:4*n*-6	0.4 ^a^	0.4 ^a^	0.3 ^a,b^	0.3 ^a,b^	0.2 ^b^	0.3	0.4	0.4	0.02	0.04	0.14	0.88
22:5*n*-3 (DPA)	2.7 ^b^	2.8 ^a,b^	3.1 ^a,b^	3.2 ^a^	3.3 ^a^	2.8 ^b^	2.9 ^b^	3.2 ^a^	0.07	0.04	0.05	0.29
22:0	1.2	1.2	1.1	1.1	1.1	1.1	1.1	1.2	0.04	0.85	0.33	0.93
23:0	0.4	0.4	0.4	0.3	0.3	0.4	0.3	0.3	0.01	0.48	0.09	0.30
24:0	1.4	1.3	1.3	1.2	1.2	1.3	1.2	1.4	0.05	0.77	0.22	0.99
∑SFA	40.5	40.5	40.0	39.4	39.6	40.4	39.7	40.3	0.26	0.70	0.64	0.94
∑MUFA	21.1	21.4	20.5	21.7	22.0	21.5	22.6	19.7	0.43	0.88	0.06	0.97
∑PUFA	38.4	38.1	39.5	38.9	38.4	38.1	37.7	40.0	0.40	0.87	0.10	0.79
PUFA/SFA	0.9	0.9	1.0	1.0	1.0	0.9	0.9	1.0	0.01	0.93	0.45	0.82
∑*n*-3 PUFA	8.7 ^b^	8.8 ^b^	9.8 ^a,b^	10.0 ^a^	10.3 ^a^	9.5	8.8	9.7	0.19	0.01	0.06	0.70
∑*n*-6 PUFA	28.2	27.5	28.0	27.1	26.1	26.7	27.1	28.7	0.38	0.49	0.22	0.86
*n*-6/*n*-3	3.3 ^a^	3.2 ^a,b^	2.9 ^a,b,c^	2.7 ^b,c^	2.5 ^c^	2.8	3.1	3.0	0.08	0.02	0.39	0.92
Content (mg/100 g wet tissue)												
18:3*n*-3	18.6	19.7	19.9	23.6	24.6	22.0	22.5	18.6	1.72	0.78	0.63	0.18
20:4*n*-6	243.6	245.0	223.2	204.3	202.7	234.5	207.0	239.6	9.35	0.46	0.66	0.48
EPA	33.7	37.6	38.1	41.7	45.5	43.0	32.3	39.9	2.00	0.39	0.17	0.86
22:5*n*-6	2.2 ^a^	2.2 ^a^	1.3 ^b^	1.5 ^a,b^	1.2 ^b^	1.8	1.6	1.9	0.13	0.02	0.82	0.67
DHA	39.2	39.2	39.3	40.5	42.1	46.5 ^a^	33.0 ^b^	40.9 ^a,b^	1.87	0.98	0.05	0.28
DPA	50.3	49.7	50.6	51.1	51.3	52.7	45.5	53.5	1.93	0.92	0.44	0.35
EPA + DHA	72.9	76.8	77.4	82.2	87.6	89.5 ^a^	65.3 ^b^	80.8 ^a,b^	3.45	0.87	0.05	0.70
EPA + DHA + DPA	123.2	126.5	128.0	133.3	138.9	142.2	110.8	134.3	5.14	0.96	0.16	0.56

^1^ All definitions and abbreviations are as indicated in Table 1 and Table 2. ^2^ FA: fatty acid; Row means bearing different superscripts within a fixed factor significantly differ (*p* < 0.05).

**Table 5:** Influence of omega–3 oil supplementation and breed on plasma metabolite profiles of prime lambs (mmol/L).

Item ^1^		Treatment				Breed			SEM	Normal Range	*p* Value		
	Control	2.5C	5C	2.5F	5F	M × M	C × M	W × C			T	B	T × B
Cholesterol	1.3	1.5	1.5	1.4	1.2	1.3	1.4	1.4	0.04	1.1–1.5	0.13	0.62	0.92
Urea	7.8	7.7	7.5	7.6	7.5	7.8	7.4	7.3	0.16	2.8−7.2	0.27	0.44	0.93
Calcium	2.6	2.6	2.7	2.6	2.7	2.7	2.7	2.6	0.02	2.4−3.2	0.26	0.15	0.61
Magnesium	1.1	1.0	1.0	1.0	1.0	1.0	1.0	1.1	0.02	0.8−1.2	0.83	0.64	0.62
BHB	0.3	0.3	0.4	0.3	0.4	0.3	0.4	0.3	0.02	0.0−0.8	0.66	0.85	0.95
Glucose	4.2	4.5	4.3	4.1	4.2	4.0 ^b^	4.3 ^a^^,^^b^	4.5 ^a^	0.07	2.8−4.5	0.48	0.03	0.04

^1^ As indicated in Table 2; BHB: Beta–hydroxybutyrate. Row means bearing different superscripts within a fixed factor significantly differ (*p* < 0.05).
